# Improvements in School Food Offerings over Time: Variation by School Characteristics

**DOI:** 10.3390/nu15081868

**Published:** 2023-04-13

**Authors:** Sarah Martinelli, Theresa Bui, Francesco Acciai, Michael J. Yedidia, Punam Ohri-Vachaspati

**Affiliations:** 1 College of Health Solutions, Arizona State University, Phoenix, AZ 85004, USA; sarah.marintelli@asu.edu (S.M.);; 2 College of Medicine Phoenix, University of Arizona, Phoenix, AZ 85721, USA; 3 Rutgers Center for State Health Policy, Rutgers Institute of Health, Health Care Policy and Aging Research, Rutgers University, New Brunswick, NJ 08901, USA

**Keywords:** nutrition policy, nutritional quality, sociodemographic disparities, school nutrition, school meal programs

## Abstract

The 2010 Healthy, Hunger-Free Kids Act (HHFKA) improved the nutritional quality of food served in schools. This longitudinal study examined school food offerings over time from school year 2010-11 to 2017-18 in public schools (n = 148) in four New Jersey cities. Six food indices were used to assess the number of healthy and unhealthy items offered as part of the National School Lunch Program (NSLP), in vending machines, and à la carte (i.e., competitive foods). Multilevel, multivariable linear regression with quadratic terms was used to model the trends over time. Interaction terms were added to examine whether the time trends varied by school-level factors, such as proportion of students eligible for free or reduced-price meals (FRPMs), race/ethnicity of enrolled students, and school level. Over the study period, healthy items offered in the NSLP increased (*p* < 0.001), while unhealthy items in the NSLP decreased (*p* < 0.001). Significantly different rates of declines in NSLP unhealthy offering were observed among schools at the two extremes of FRPM eligibility (*p* < 0.05). The trends for healthy and unhealthy foods offered in competitive foods showed significant non-linear trends, and differences were observed for school-level race/ethnicity, with worse outcomes for schools with majority Black student enrollment.

## 1. Introduction

The vast majority of American children have diets that do not meet the Dietary Guidelines for Americans, exceeding recommended amounts of sodium, sugar, and saturated fats while falling short on fruits and vegetables [1,2,3]. In addition, there are marked socioeconomic status (SES) and racial/ethnic disparities in food consumption among children, with children from lower SES and non-White households typically consuming less healthy diets [4,5]. Unhealthy dietary behaviors have been shown to be associated with childhood obesity that persists into adulthood [6,7,8]. Obesity rates among school-age children in the United States continue to be a major public health concern. Based on data from 2017–2020, 19.7% of all 2- to 19-year-old children had obesity, and rates were even higher among Hispanic children (26.2%), non-Hispanic Black children (24.8%), and children from the lowest-income group (25.8%) [9]. Improving children’s diet is crucial (1) for addressing this public health risk and (2) for combating health disparities at the population level.

Schools are well positioned to shape children’s diets, as almost all children in the United States attend public or state-accredited private schools, where they spend on average between 6 and 7 h per day, most days of the year [10]. For students who consume both school breakfast and lunch, school meals can provide more than half (58%) of their daily calories [10]. The School Breakfast Program (SBP) and National School Lunch Program (NSLP)—the two signature school meal programs of the United States Department of Agriculture (USDA)—are offered in almost 100,000 schools in the United States [11]. Meals offered through the NSLP and SBP must meet specific nutritional standards set by the USDA to receive reimbursement. The USDA also provides guidance on foods and beverages sold in vending machines and as à la carte options outside of the school meal programs, also known as “competitive” foods and beverages.

The most recent updates to the school food guidelines were undertaken as part of the Healthy, Hunger-Free Kids Act (HHFKA) of 2010, which aligned the school nutrition standards with the Dietary Guidelines for Americans [1,12,13,14]. These updates were implemented in a stepwise fashion with regulations impacting NSLP starting in school year (SY) 2012-13, SBP in SY 2013-14, and, lastly, competitive foods (known as Smart Snacks Standards) in SY 2014-15 [15]. Since the implementation of the HHFKA, the nutritional quality of school meals and à la carte options has improved significantly, and 98.5% of all participating districts met the guidelines as of December 2015 [16,17,18,19,20,21]. There is increasing evidence that nutrition policies for foods served in schools positively influence student selection and consumption of fruits, vegetables, and whole grains [22,23,24,25,26,27,28,29,30,31]. Notably, nutrition policies requiring healthier offerings are also associated with a lower prevalence of obesity, especially among children from lower-income families [32]. 

The policies set out in the HHFKA have resulted in healthier school meals overall; however, some studies have noted differences in the types of items served, depending on school characteristics [17,18]. Bardin et al. (2020) found that the overall nutritional quality of school lunches was similar across school characteristics, such as school poverty level and the racial/ethnic composition of students. The authors also assessed the availability of competitive foods in schools and found that competitive foods offerings were less prevalent in high-poverty, majority Black, and majority Hispanic schools compared to majority White schools [33]. While there are several studies highlighting meal improvements after HHFKA, most do not examine differences by school-level race or income, and none examine these changes over time. Given the variation of when and where school-based nutrition policies were implemented over time, and prior evidence of disparities in access to healthy foods across different racial and socioeconomic groups, examination of trends in food and beverages offered in schools is warranted. This descriptive analysis examines foods offered in schools, including as part of the NSLP, in vending machines, and à la carte, from SY 2010-11 through 2017-18. This analysis describes changes in foods offered in schools over time with respect to school level (elementary vs. middle/high school), the proportion of students eligible for free or reduced-price meals (FRPMs), and the racial/ethnic composition of the school.

## 2. Materials and Methods

This study is a secondary analysis of data from the New Jersey Child Health Study (NJCHS), a longitudinal study examining the impact of food and physical activity environments on children’s weight status. A fuller description of study goals and methods is available elsewhere [16]. The current analysis uses data collected on the foods and beverages available in all K-12 public schools in four study cities: Camden, New Brunswick, Newark, and Trenton. The study was deemed exempt by the Arizona State University and Rutgers University Institutional Review Boards. 

**Availability of Food Items.** To capture the food offerings in schools, a survey using questions based on prior research was sent to all public schools in each study city [34,35,36,37,38]. The school nurse, with the assistance of the school food service professional, answered questions about foods offered during school meals as part of the NSLP, à la carte, and in vending machines. Nurses could respond via a paper survey or an online survey using the Qualtrics© (Provo, UT, USA) survey platform. Data on school food offerings were collected over an eight-year period, from SY 2010-11 through 2017-18. Surveys were fielded in SYs 2012-13, 2015-16, and 2017-18. For each round of data collection, nurses were asked questions about food items offered in their school during the current and prior school year(s). Data were provided by 127, 110, and 108 schools in the three survey rounds, respectively. The number of schools changed from year to year due to non-participation or schools not being open during a specific survey period. Overall, 80 schools contributed data for all three rounds, covering the entire study period, 37 schools contributed to two rounds, and 31 schools contributed to only one round of data.

Surveys collected information on the availability of common healthy and unhealthy offerings at three food venues: NSLP, vending, and à la carte. These data were then summarized into six indices by summing the number of healthy and unhealthy items for each venue. NSLP healthy index scores ranged from 0 to 9 and included items such as fresh fruits, whole grains, and salad bars. NSLP unhealthy index scores ranged from 0 to 5 and included items such as French fries, traditional pizza recipes (i.e., not whole grain/whole grain-rich), and dessert. À la carte healthy index scores ranged from 0 to 9 and included items such as bottled water, salad bar, and raw fruits or vegetables, whereas à la carte unhealthy ranged from 0 to 12 and included items such as soda, cookies, or salty snacks. Vending healthy and unhealthy scores ranged from 0 to 4 and from 0 to 9, respectively, and included options similar to those of à la carte. À la carte and vending machine offerings represented competitive foods available in schools. Milk is present in both the healthy and unhealthy indices based on the fat content of the milk served. This mirrors the USDA nutrition guidelines for breakfast and lunch, which require lower fat levels for flavored milk. A complete list of items in these six indices is listed in Supplementary Table S1. Additional information about the school environment survey and the development of the school food indices can be found elsewhere [16]. 

**School Characteristics**. Data from the National Center for Education Statistics (NCES) were assembled on school-level factors, such as total student enrollment, racial/ethnic composition of enrolled students, and proportion of students eligible for free or reduced-price meals for each year [39]. School race/ethnicity was classified into three categories based on the majority (>50%) race/ethnicity of enrolled students. The three categories were majority Hispanic, majority non-Hispanic Black, and majority non-Hispanic White. If the school did not have greater than 50% of any race/ethnicity, it was classified as “other”. Given the low number of schools classified as other and as majority non-Hispanic White schools (about 3% of the sample), these two categories were combined. The proportion of students eligible for FRPMs was divided into tertiles. Based on the grades offered, schools were classified into elementary and secondary (middle and high) schools. Middle and high schools were combined because only 10 schools in our sample offered the traditional middle school grades—6th, 7th, or 8th grades only. A vast majority of elementary schools included the traditional middle school grades, with 75% of the elementary schools including pre-K/K-8 schools. Finally, a binary variable indicating a school’s participation in the Community Eligibility Provision (CEP) for each year over the course of the study was obtained from the New Jersey Department of Agriculture, the state agency that oversees school meals, via an Open Public Records Act request and was used as a control variable. 

**Data Analysis.** All analyses were run in Stata version 15, and statistical significance was set at *p* < 0.05. Multilevel linear regression (command *mixed*) was used to model the number of healthy and unhealthy items served in each of the three venues to describe the school food environment over the study period, while accounting for repeated measures and the clustering of schools at the city level. All models controlled for school-level factors, including total student enrollment, majority race/ethnicity, the proportion of students eligible for FRPMs, and school level (elementary vs. secondary schools). Time was entered as a continuous variable. Specifically, models for NSLP (both healthy and unhealthy) included a linear trend for time, while models for vending and à la carte (both healthy and unhealthy) also included a quadratic term to better reflect the observed time trends.

To examine if the trends observed in the full sample differed by school-level factors, the models were expanded by adding, one at a time, a series of interaction terms between time and (1) race/ethnicity (majority Hispanic vs. majority Black), (2) proportion of students eligible for FRPMs (highest tertile schools vs. lowest tertile schools), and (3) school level (elementary vs. secondary schools). The interaction analysis focused on majority Hispanic and majority Black schools due to the small sample size of schools that were classified as non-Hispanic White/other; additionally, to better see trends at the extremes of the income spectrum, only the highest and lowest tertiles of FRPM eligibility were compared.

## 3. Results

### 3.1. Descriptive Results

Table 1 presents descriptive statistics and school-level characteristics of the sample at the beginning (SY 2010-11) and end of the study period (SY 2017-18). In SY 2010-11, the study sample included 127 K-12 public schools with a mean enrollment of 529 students. 

Most of the study schools were majority Hispanic (45.7%) or majority non-Hispanic Black (50.4%). Two-thirds of these schools (67.7%) were elementary schools, and one-third (32.3%) were middle or high schools. When examining the overall sample across all school years (Table 2), there were no associations between the number of healthy or unhealthy items served in NSLP meals and total student enrollment, the proportion of students eligible for FRPMs, or school level (elementary vs. secondary schools). However, both the number of healthy items and the number of unhealthy items served in the NSLP differed by race/ethnicity of enrolled students. Schools with majority Hispanic students served fewer healthy (−0.17, *p* = 0.049, CI: −0.34–0.00) and unhealthy (−0.41, *p* < 0.001, CI: −0.65–−0.18) items compared to schools with majority non-Hispanic Black students. Schools with majority non-Hispanic White/other students served fewer NSLP unhealthy items compared to schools with majority non-Hispanic Black students (−0.42, *p* < 0.001, CI: −0.64–−0.21).

School level was significantly associated with the number of healthy and unhealthy items served both in vending machines and à la carte. In all cases, middle/high schools served more items (Table 2). Schools with a higher percentage of students eligible for FRPMs served more unhealthy à la carte items compared to schools with fewer students eligible for FRPMs (0.27; *p* = 0.029; CI: 0.05–0.52). Finally, the unhealthy vending index was associated with school size, where larger schools were more likely to serve more unhealthy vending items (0.019, *p* = 0.01, CI: 0.01–0.11).

### 3.2. Overall Trends

**Figure 1:**
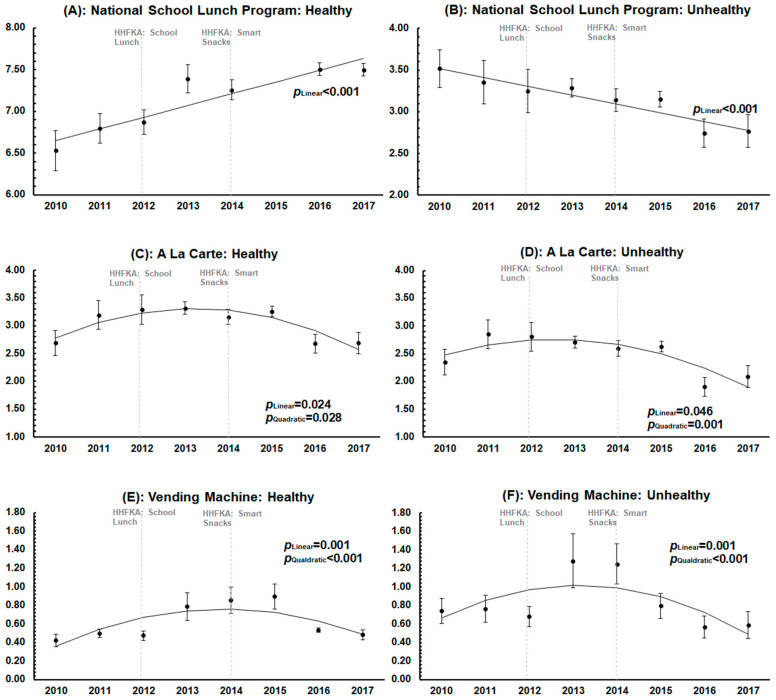
Year-by-year point estimates and predicted trends of each of the six food environment indices from school year 2010-11 to 2017-18 in the full sample. Dots represent the adjusted point estimates with confidence intervals estimated from the multilevel multivariable linear regression model. Lines represent the predicted line of best fit over the study period. The 2012 vertical line represents the implementation of the Healthy Hunger-Free Kids Act (HHFKA) guidelines for NSLP that occurred in 2012. The 2014 vertical line represents the implementation of the Smart Snacks guidelines (within the HHFKA) that occurred in 2014.

Figure 1A–F presents the results from the multilevel multivariable regression analyses, displaying predicted mean scores for all indices from SY 2010-11 to SY 2017-18. The number of healthy items in the NSLP showed a positive linear trend over the study period (*p* < 0.001), with the average number of items offered increasing from 6.5 to 7.5. The number of unhealthy items in the NSLP showed a negative linear trend (*p* < 0.001) over the same time period, with the average number of items offered decreasing from 3.5 to 2.8. Trends in à la carte and vending machines, for both healthy and unhealthy items, showed a significant quadratic pattern of change: increasing after 2012, which would align with the implementation of updated nutrition guidelines in the HHFKA, and decreasing in 2014, again aligning with new nutrition guidelines in Smart Snacks.

### 3.3. Trends by School-Level Characteristics

**Figure 2:**
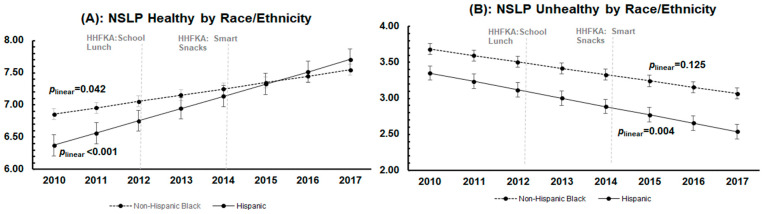

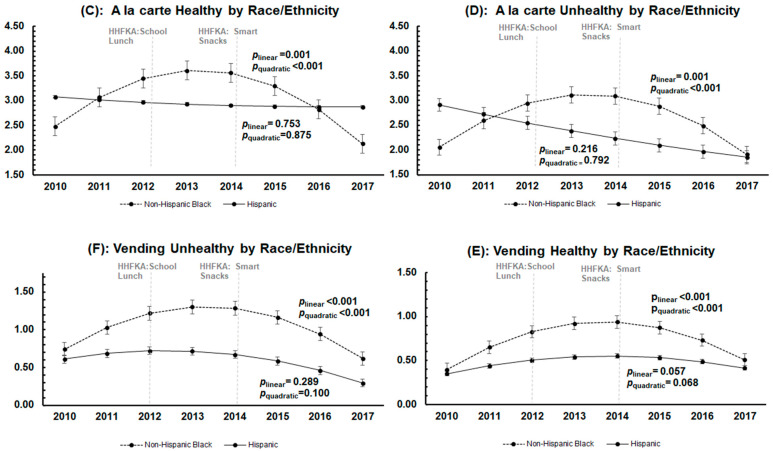
Year-by-year point estimates and predicted trends of each of the six food environment indices from school year 2010-11 to 2017-18 by race/ethnicity. Dots represent the adjusted point estimates with confidence intervals estimated from the multilevel multivariable linear regression model. Lines represent the predicted line of best fit over the study period. The 2012 vertical line represents the implementation of the Healthy Hunger-Free Kids Act (HHFKA) guidelines for NSLP that occurred in 2012. The 2014 vertical line represents the implementation of the Smart Snacks guidelines (within the HHFKA) that occurred in 2014.

Figure 2A–F presents trends by race/ethnicity of enrolled students in foods offered in NSLP, à la carte, and vending over the study period. Figure 2A shows that the trends in NSLP healthy in schools with majority Hispanic and non-Hispanic Black students mirrored that of the full sample (i.e., positive in both groups) and that these trends were not significantly different from each other (*p* = 0.06). For NSLP unhealthy, Figure 2B shows that the trend for each group was negative, similar to the overall trend; however, in schools with majority Hispanic students, the trend was significant (*p* = 0.004), while the trend for schools with majority non-Hispanic Black students was not (*p* = 0.125). Trends by race/ethnicity for both healthy and unhealthy à la carte indices were statistically different from each other (*p* < 0.001 and *p* = 0.008; Figure 2C,D). Schools with majority non-Hispanic Black students showed a significant quadratic trend in the availability of both healthy and unhealthy foods offered à la carte (*p* linear < 0.001 and *p* quadratic = 0.001 for both healthy and unhealthy), mirroring the overall sample trends. In contrast, trends in both healthy and unhealthy foods offered à la carte in schools with majority Hispanic students were approximately flat. Similar to à la carte, the trends in vending healthy were different by race (*p* linear = 0.001 and *p* quadratic = 0.001 for à la carte healthy and *p* linear 0.005 and *p* quadratic = 0.008 for à la carte unhealthy). In Figure 2E, the trend in schools with a majority non-Hispanic Black students had a significant quadratic component mirroring the full sample (*p* < 0.001), while neither the linear nor the quadratic component was different from 0 in schools with majority Hispanic students (*p* = 0.057 and *p* = 0.068, respectively). Finally, for unhealthy vending shown in Figure 2F, trends by race/ethnicity were not statistically different from one another (*p* linear = 0.053 and *p* quadratic = 0.077). Schools with majority non-Hispanic Black students had statistically significant quadratic trends, which mirrored the quadratic trend of the full sample, while the trend for schools with majority Hispanic students was approximately flat.

**Figure 3:**
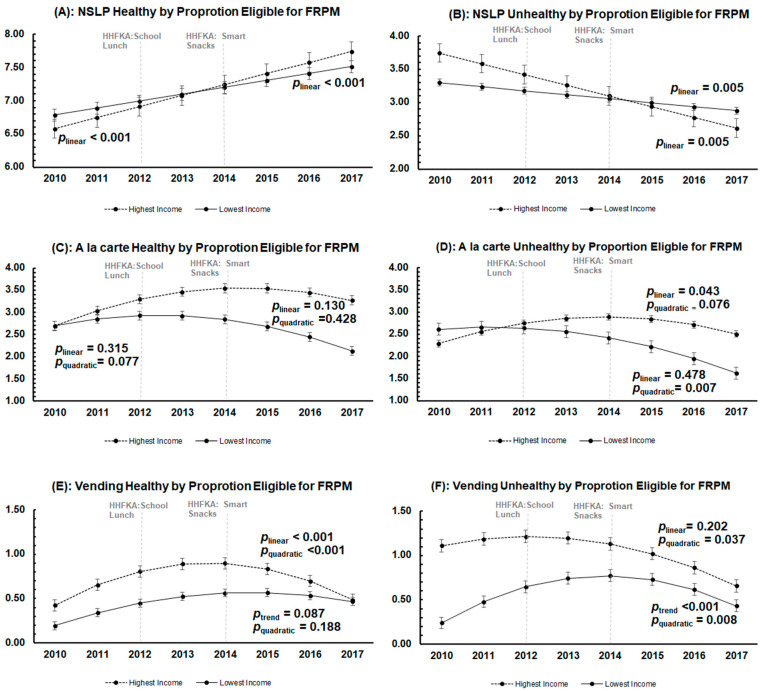
Year-by-year point estimates and predicted trends of each of the six food environment indices from school year 2010-11 to 2017-18 by proportion of students eligible for FRPMs. Dots represent the adjusted point estimates with confidence intervals estimated from the multilevel multivariable linear regression model. Lines represent the predicted line of best fit over the study period. The 2012 vertical line represents the implementation of the Healthy Hunger-Free Kids Act (HHFKA) guidelines for NSLP that occurred in 2012. The 2014 vertical line represents the implementation of the Smart Snacks guidelines (within the HHFKA) that occurred in 2014.

Figure 3A–F presents results from analysis examining differences in trends by the proportion of students eligible for FRPMs, where schools from the highest and lowest tertiles of FRPMs are compared. The trends in NSLP healthy and unhealthy in schools with the highest and lowest proportion of students eligible for FRPMs were significant and reflected trends in the overall sample—positive linear for NSLP healthy and negative linear for NSLP unhealthy (Figure 3A,B). However, for NSLP unhealthy, there were significant differences in the trends between the two income groups (*p* = 0.015), with higher-income schools showing a steeper decline over the study period than lower-income schools. For à la carte healthy, the trend was not significant for both groups, and the trend lines were not different from each other (Figure 3C). The trend in la carte unhealthy, however, was different by student eligibility for FRPMs (*p* linear = 0.039 and *p* quadratic = 0.477) (Figure 3D). The lowest-income schools had a significant decrease in the number of unhealthy à la carte items served over time (*p* quadratic = 0.007), while the number of unhealthy à la carte items was approximately stable in higher-income schools. For vending healthy, the trends between the highest- and lowest-income schools were not significantly different from one another (*p* linear = 0.423 and *p* quadratic = 0.282); however, the trend in the higher-income schools did show a significant change (i.e., an initial upward trajectory followed by a decline) in the number of healthy items offered in vending machines over time (*p* < 0.001) (Figure 3E). Finally, the number of unhealthy items offered in vending machines in the highest-income schools tended to decline over time, while the trend for the lowest-income school slightly increased at first and then also declined (Figure 3F).

**Figure 4:**
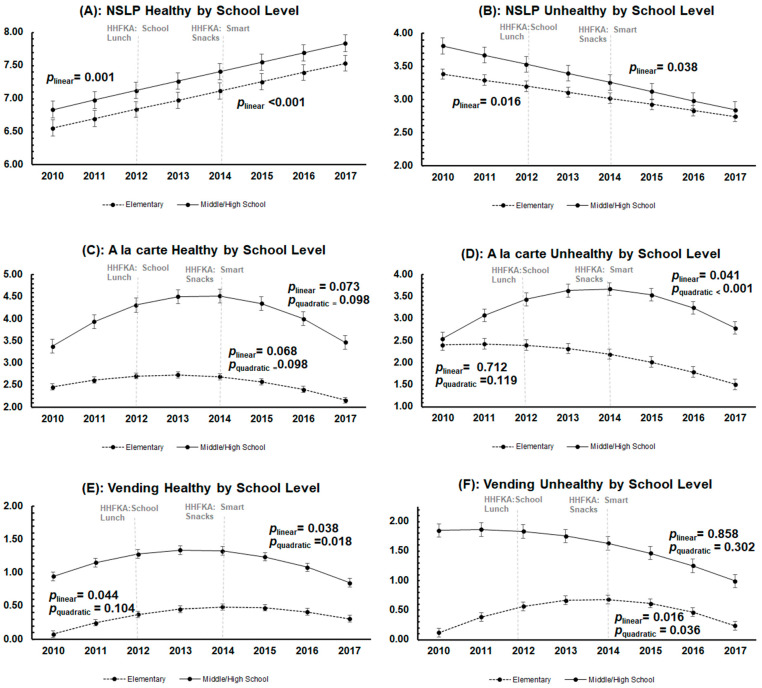
Year-by-year point estimates and predicted trends of each of the six food environment indices from school year 2010-11 to 2017-18 by school level. Dots represent the adjusted point estimates with confidence intervals estimated from the multilevel multivariable linear regression model. Lines represent the predicted line of best fit over the study period. The 2012 vertical line represents the implementation of the Healthy Hunger-Free Kids Act (HHFKA) guidelines for NSLP that occurred in 2012. The 2014 vertical line represents the implementation of the Smart Snacks guidelines (within the HHFKA) that occurred in 2014.

When stratified by school level, trends in the availability of foods in each of the three venues approximately mirrored those of the full sample for both elementary and secondary schools (Figure 4A–F). Trends did not vary by school level for any of the indices.

## 4. Discussion

This study described the change in the availability of food and beverage items offered in various school venues (i.e., school lunch, à la carte, and vending machines) over time and by school-level factors, such as race/ethnicity, proportion of students eligible for FRPMs, and grade level, in a sample of urban schools across four cities in New Jersey. Overall, study results show that school food offerings in NSLP and competitive foods changed significantly over time and the timing of these changes aligned with major federal policy initiatives, such as the implementation of guidelines for reimbursable school meals and competitive foods. These changes in food offering over time did not affect all schools in the same way; significant differences in these trends were noted for race/ethnicity of enrolled students and for the proportion of students eligible for FRPMs.

Consistent with previous research, the current study found improvements in the school food offerings over time as shown by a steady increase in the number of healthy items offered in the NSLP and a concurrent decrease in unhealthy NSLP items, which is likely to have occurred in response to the implementation of the HHFKA [23,40]. These trends suggest that national nutrition policies can impact student health through measurable changes in the foods offered in school meals. While health outcomes were not measured as part of this specific study, several studies have connected the improved school food environment post-HHFKA to improved nutrient intake and lower BMI among participating students [28,29,31,41]. Overall trends in the competitive food offerings changed in a non-linear fashion with an increase in both healthy and unhealthy items sold in both venues occurring just after the implementation of the HHFKA in 2012, followed by a decrease in the number of items offered in 2014 around the implementation of the Smart Snacks standards. The quadratic, reverse-U-shaped, pattern seen in the current study is interesting and might reflect the initial shift of foods out of the school lunch program (which were no longer compliant due to the HHFKA nutrition guidelines) into competitive food venues, where they were still compliant in 2012 and 2013. This trend then reversed in 2014 after the implementation of the Smart Snacks standards, when similar nutrition guidelines were placed on competitive foods. While the authors are not aware of other studies that have examined this specific trend, the current results suggest that schools are responsive to nutrition guidelines. These results also highlight the need for consistent guidelines across the school campus to ensure that unhealthy foods are not simply moved from one venue to another but instead that schools are providing an environment that promotes healthy dietary choices campus-wide. 

When examined by school-level race/ethnicity, the current study found that schools with a majority of students who are Hispanic and non-Hispanic White/other served fewer unhealthy items in the NSLP compared to schools with a majority of non-Hispanic Black students and these differences remained over the course of the study period. This finding runs contrary to cross-sectional results described by Bardin and colleagues (Bardin 2020), which saw no difference in the nutritional quality of school meals as defined by the total Healthy Eating Index (HEI) scores using SY 2014-15 meal quality data from a nationally representative sample of schools as part of the School Nutrition and Meal Cost Study. The study did report some differences in individual HEI component scores by race/ethnicity, but overall there was no difference in the nutritional quality of school meals as defined by the total HEI scores [33]. In addition to differences in foods offered in the NSLP, the trend in foods offered in both competitive food venues (à la carte and vending machines) also varied by race; in schools with majority non-Hispanic Black students, the availability of foods in these venues increased immediately after the HHFKA and then started to fall after Smart Snacks, while schools with majority Hispanic students did not see significant changes in à la carte offerings—healthy or unhealthy—over time.

Across all levels of income, schools increased the availability of healthy items in the NSLP over time at similar rates in the years after the implementation of the HHFKA. In contrast, while both the highest- and lowest-income schools had significant negative trends in the number of unhealthy items offered in school lunch meals, the decline in higher-income schools was steeper than that observed for lower-income schools. Given that higher-income schools in this sample were serving more unhealthy items in the NSLP at the start of the study period, the steeper rate of change might reflect the overall greater need for improvement in these schools to meet the updated guidelines. Higher-income schools offered more competitive foods and beverages, which might be due to the higher number of students who are not eligible for free or reduced-price meals and who are more likely to buy items in competitive food venues rather than, or in addition to, NSLP meals. School food departments are more likely to offer a greater number of competitive foods if there is a demand for those foods. This is in line with the current finding showing a different trend in the number of à la carte healthy items across school-level income, with an overall higher number of items in higher-income schools. Given that à la carte foods, even after stricter standards imposed by Smart Snacks policies, tend to be less nutritious compared to complete NSLP meals, the increased presence of these foods in certain schools might reduce the otherwise positive impact that NSLP meals have on food offering and overall diet quality. These findings also highlight that all schools, regardless of income and total number of reimbursable meals served, can benefit from the nutrition guidelines set out in the HHFKA.

A key strength of the current study is its longitudinal data collection, which allowed for a review of trends over time as policies changed. Another strength, its focus on schools that serve predominantly low-income and minority student populations, is accompanied by related limitations: The analysis of trends by race was limited by the fact that the sample did not include sufficient numbers of schools with majority non-Hispanic White/other. This limited our ability to compare trends in the school food environment across all three major racial groups. Further, the majority of the schools in the sample had high FRPM rates, so schools that were classified as having a lower proportion of students eligible for FRPMs still had approximately 60% of students qualifying for free meals. It is possible that a more diverse sample (both by race/ethnicity and FRPM eligibility) might have produced different results. 

## 5. Conclusions

The school food environment plays an important role in helping students develop healthy dietary habits. Our results suggest that school food and beverage availability over time is responsive to policy changes. Schools in this study showed significant improvements in NSLP offerings after the implementation of HHFKA and in the à la carte and vending offerings after the implementation of the Smart Snacks guidelines. However, these changes were not experienced equally by students in all schools, suggesting differential uptake of policies. Healthy school environments are a cornerstone of public health approaches for improving the diet quality of school-age children, and some schools might benefit from additional support to fully implement policies aimed at improving these environments. This support could come from the USDA, in the form of training or equipment grants, or from school- and district-level committees focused on the implementation and sustainability of food-related policies. Additionally, school food departments are responsible for the implementation and monitoring of the policies outlined in the HHFKA. However, there are many other school-level policies that can impact meal service and the food available to students, such as the time and duration of lunch service or the availability of vending machines, that are not under the purview of these departments. Promoting healthy food environments when planning the school day and developing school-wide policies could help maximize the effectiveness of federal and state policies aimed at improving student health. Future research should investigate additional strategies for ensuring equitable implementation of policy so all students can benefit from improvements in school food environments. 

## Figures and Tables

**Table 1:** Demographic characteristics of public schools from four New Jersey school districts included in the analytical sample in 2010 (baseline) and 2017.

	2010	2017
	Mean (SD) Freq (%)	Mean (SD) Freq (%)
	n = 127	n = 108
Total Student Enrollment Mean (SD)	529 (302.3)	677 (404.8)
School Majority Race/Ethnicity n (%)		
Hispanic	58 (45.7)	60 (55.5)
Non-Hispanic White/Other	5 (3.9)	3 (2.7)
Non-Hispanic Black	64 (50.4)	45 (41.7)
Proportion of students eligible for FRPMs (mean %)		
Lowest Tertile or Highest Income	66.3	58.1
Middle Tertile or Middle Income	82.6	81.4
Highest Tertile or Lowest Income	93.2	91.0
School Level n (%)		
Elementary	86 (67.7)	73 (67.6)
Middle/High School	41 (32.3)	35 (32.4)
Participation in Community Eligibility Provision n (%)		
Yes	0 (0.0)	29 (26.9)
No	127 (100)	79 (73.3)
City n (%)		
Camden	27 (21.3)	15 (16.7)
Newark	14 (11.0)	11 (12.2)
New Brunswick	65 (51.2)	49 (54.4)
Trenton	21 (16.5)	15 (16.7)

**Table 2:** Association between each index (NSLP healthy and unhealthy, à la carte healthy and unhealthy, vending healthy and unhealthy) and school-level factors (total student enrollment, majority race/ethnicity, proportion of students eligible for FRPMs, and school level) over the study period (2010-11 to 2017-18).

	NSLP Healthy		NSLP Unhealthy		À La Carte Healthy		À La Carte Unhealthy		Vending Healthy		Vending Unhealthy	
	Coef	95% CI	Coef	95% CI	Coef	95% CI	Coef	95% CI	Coef	95% CI	Coef	95% CI
Total Student Enrollment	0.18	(−0.03, 0.06)	−0.00	(−0.06, 0.06)	0.03	(−0.06, 0.12)	0.03	(−0.07, 0.13)	0.02	(0.00, 0.04)	0.06 **	(0.01, 0.11)
Majority Race/Ethnicity												
Non-Hispanic Black	Reference		Reference		Reference		Reference		Reference		Reference	
Hispanic	−0.17 *	(−0.34, −0.00)	−0.41 ***	(−0.65, −0.18)	0.14	(−1.20, 1.49)	−0.17	(−1.51, 1.18)	−0.07	(−0.30, 0.14)	−0.23	(−0.67, 0.22)
Non-Hispanic White/Other	0.10	(−0.21, 0.41)	−0.42 ***	(−0.64, −0.21)	0.50	(−0.94, 1.94)	0.11	(−1.61, 1.82)	−0.10	(−0.72, 0.52)	−0.24	(−0.89, 0.40)
Proportion of students eligible for FRPMs												
Lowest Tertile/Highest Income	Reference		Reference		Reference		Reference		Reference		Reference	
Middle Tertile/Middle Income	0.02	(−0.09, 0.13)	0.06	(−0.22, 0.34)	0.12	(−0.34, 0.63)	0.06	(−0.22, 0.34)	0.05	(−0.11, 0.20)	−0.12	(−0.34, 0.09)
Highest Tertile/Lowest Income	0.15	(−0.16, 0.47)	0.03	(−0.23, 0.29)	0.10	(−0.61, 0.80)	0.27 *	(0.05, 0.52)	0.01	(−0.03, 0.52)	−0.13	(−0.43, 0.17)
School Level	0.25	(−0.19, 0.68)	0.19	(−0.13, 0.51)	1.5 ***	(0.80, 2.20)	1.04 **	(0.24, 1.84)	0.74 ***	(0.64, 0.85)	0.90 ***	(0.41, 1.40)

* *p* < 0.05; ** *p* < 0.01; *** *p* < 0.001.

## Data Availability

The data presented in this study are available upon request from the corresponding author.

## Supplementary Materials

The following supporting information can be downloaded at: https://www.mdpi.com/article/10.3390/nu15081868/s1, Table S1: List of food items included in the six indices to capture school food environments in K-12 schools.
