# Supplementary material for: Improvements in School Food Offerings over Time: Variation by School Characteristics
Source: Nutrients. 2023 Apr 13;15(8):1868. doi: 10.3390/nu15081868 (PMC10146944; doi:10.3390/nu15081868)
Supplement: Supplementary file 1 [file nutrients-15-01868-s001.zip › nutrients-2294754-supplementary.pdf]

Table S1: List of food and beverage items included in the six indices capturing different elements of the school food environment.

Index name and score range	Items Included	
**NSLP Healthy Items (0-9)**	At least Half Whole Grains	Fat-free Flavored Milk
	Whole Grains	Fresh Fruit
	Variety of Vegetables	Raw Vegetables
	Modified Pizza (whole grain rich crust and/or low-fat cheese)	Salad Bar
	Fat-free / 1% Milk	
**NSLP Unhealthy Items (0-5)**	Fries	
	Pizza (traditional recipe)	
	Dessert	
	Full fat / 2% Milk	
	Full / 1% Flavored Milk	
**Vending Machine Healthy Items (0-4)**	Bottled Water	
	100% Juice	
	Fat-free / 1% Unflavored Milk	
	Fat-free Flavored Milk	
**Vending Machine Unhealthy Items (0-9)**	Juice Drink	Full fat / 2% / 1% Flavored Milk
	Diet Soda	Salty snacks
	Soda	Cookies, cakes
	Energy or Sports Drinks	Candy
	Full fat / 2% Unflavored Milk	
**A La Carte Healthy Items (0-9)**	Bottled Water	Raw Fruit and vegetables
	100% Juice	Salad Bar
	Fat-free / 1% Unflavored Milk	Sandwiches
	Fat-free Flavored Milk	Modified Pizza (whole grain rich crust and/or low-fat cheese)
	Dairy Foods, Lower in Fat	
**A La Carte Unhealthy Items (0-12)**	Juice Drinks	Salty Snacks
	Diet Soda	Fried Potatoes
	Soda	Pizza (traditional recipe)
	Energy or Sports Drinks	Cookies, Cakes
	Full fat / 2% Unflavored Milk	Frozen Desserts
	Full fat / 2% / 1% Flavored Milk	Candy
